# Comparative genomics revealed drastic gene difference in two small Chinese perches, *Siniperca undulata* and *S. obscura*

**DOI:** 10.1093/g3journal/jkad101

**Published:** 2023-05-09

**Authors:** Liang Lu, Junlong Jiang, Jinliang Zhao, Chenhong Li

**Affiliations:** Shanghai Universities Key Laboratory of Marine Animal Taxonomy and Evolution, Shanghai Ocean University, Shanghai 201306, China; Engineering Research Center of Environmental DNA and Ecological Water Health Assessment, Shanghai Ocean University, Shanghai 201306, China; Shanghai Universities Key Laboratory of Marine Animal Taxonomy and Evolution, Shanghai Ocean University, Shanghai 201306, China; Engineering Research Center of Environmental DNA and Ecological Water Health Assessment, Shanghai Ocean University, Shanghai 201306, China; Key Laboratory of Exploration and Utilization of Aquatic Genetic Resources, Ministry of Education, Shanghai Ocean University, Shanghai 201306, China; Shanghai Collaborative Innovation for Aquatic Animal Genetics and Breeding, Shanghai Ocean University, Shanghai 201306, China; Shanghai Universities Key Laboratory of Marine Animal Taxonomy and Evolution, Shanghai Ocean University, Shanghai 201306, China; Engineering Research Center of Environmental DNA and Ecological Water Health Assessment, Shanghai Ocean University, Shanghai 201306, China

**Keywords:** Sinipercidae, genome assembly, positive selection, gene families, population history

## Abstract

*Siniperca undulata* and *S. obscura* (Centrarchiformes: Sinipercidae) are small Chinese perches, living in creeks and streams in southern China. While they have sympatric distribution and occupy similar macrohabitat, their body sizes and ecological niches have many differences. Determining the genome sequences of *S. undulata* and *S. obscura* would provide us an essential data set for better understanding their genetic makeup and differences that may play important roles in their adaptation to different niches. We determined the genome sequences of both *S. undulata* and *S. obscura* using 10× genomics technology and the next-generation sequencing. The assembled genomes of *S. undulata* and *S. obscura* were 744 and 733 Mb, respectively. Gene family analysis revealed that there were no overlap between *S. undulata* and *S. obscura* in terms of rapid expanding and rapid contracting genes families, which were related to growth, immunity, and mobility. Positive selection analyses also cooperated that the function of selected genes involve growth, athletic ability, and immunity, which may explain the preference of different niches by *S. undulata* and *S. obscura*. Pairwise sequentially Markovian coalescent analyses for the two species suggested that populations of both *S. undulata* and *S. obscura* showed a rising trend between 90 and 70 Ka probably due to the mild environment during the last interglacial period. A stage of population shrinking occurred from 70 to 20 Ka, which was in with the Tali glacial period in eastern China (57–16 Ka).

## Introduction

*Siniperca undulata* and *S. obscura* are sister species belonging to the Centrarchiformes: Sinipercidae, which are freshwater carnivorous fishes endemic to China. They are closely related to the well-known North American sunfish (Centrarchidae) and has a very important role in the ecological health of streams and rivers in southern China.

*Siniperca undulata* and *S. obscura* are mainly distributed in rivers and lakes in plain areas below 300 m in elevation, and have sympatric distribution and occupy similar macrohabitat. *Siniperca undulata* is mainly distributed in the Oujiang River, tributaries south of the Yangtze River, parts of the Pearl River basin, and tributaries of the Qiantang River, whereas *S. obscura* is mainly distributed in tributaries south of the Yangtze River (Li 1991). The average body length of *S. undulata* is 10–15 cm, and the body shape is oblong with several yellow ripples on the side of the body. *Siniperca obscura* has a body length of 6–12 cm, a laterally flattened body form, and dark body color (Nichols 1943; Hwang *et al.* 1988).

Phenotypic differences between *S. undulata* and *S. obscura* may arise as they adapt to different ecological niches. *Siniperca undulata* and *S. obscura* differ markedly in body size, which may be accompanied by differences in growth and metabolic rates (Brown *et al.* 2004), as well as having different effects on population size (White *et al.* 2007). Typically, larger body size species reproduce more slowly. In *S. undulata*, which are larger and have a minimum population doubling time <15 months, and *S. obscura*, which is smaller but has a minimum population doubling time in 1.4–4.4 years (Rainer *et al.* 2016). In addition, the yellow ripples on the side of the body of *S. undulata* may provide camouflage coloration for its activity in streams or shallower water layers, while the darker body coloration of *S. obscura* may provide its more benthic habits.

Current studies have paid less attention to *S. undulata* and *S. obscura*, with only a few phylogenetic studies, or studies for developing molecular markers (Chen *et al.* 2010; Song *et al.* 2017), which contain very limited genetic information, mostly focused on mitochondrial genome (Zhao *et al.* 2006). For Sinipercidae, recent studies have also focused on aquaculture, breeding, and immunization, less common on genomes of species with high economic value, such as *S. chuatsi*, *S. knerii*, and *S. scherzeri* (He *et al.* 2020; Lu and Zhao 2020a). For *S. undulata* and *S. obscura*, genome-wide data have never been reported, which hampered us from understanding molecular pathway under phenotypic differences of these two species and their adaptive values to divergent ecological niches.

Here, we used 10× genomic technology to sequence and assemble whole genomes of *S. undulata* and *S. obscura* at low cost. This can provide fundamental data for comparative studies of Centrarchiformes, especially Sinipercidae. We have further compared genomes of *S. undulata* and *S. obscura* to provide new insights into the genetic basis differences in growth, immunity, and motility. These genomes further complement the data base *Siniperca* research and offer new possibilities not only for in-depth studies of fish evolution and biological functions but also for providing additional genetic resources needed for breeding in this economically important taxon.

## Materials and methods

### Sample collection and extraction, amplification of genomic DNA

**Fig. 1.**
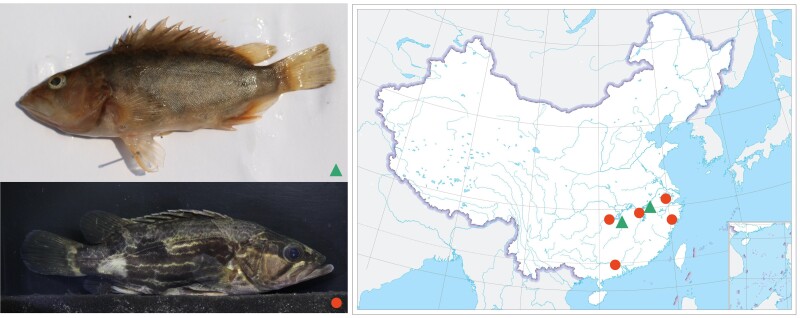
Samples collected from Jingdezhen, Jiangxi Province of China. The dots and triangles in the map (right) show their distribution areas of *S. undulata* (bottom left) and *S. obscura* (top left), respectively.

Both samples of the *S. undulata* and *S. obscura* were collected from Jingdezhen, Jiangxi Province of China in 2019 June 3 (Fig. 1). Muscle tissue was taken and then kept at −80°C. DNA was extracted within 48 h using a modified CTAB protocol (Dellaporta *et al.* 1983).

To get high molecular weight DNA (HMW, DNA size greater than 50 kb) for 10× genomics library, the extracted DNA was processed with magnetic beads based on Monarch HMW DNA Extraction Kit: Tissue Protocol. The purified large fragments were kept in $-20^{\circ }$.

### Illumina Hiseq × Ten sequencing, denovo genome assembly and quality evaluation

DNA libraries for 10× genomics system were constructed using 0.18 ng of HMW DNA. The DNA were put in the microfluid to react with barcodes and the 8-bp primers. Subsequently, gel beads were broken and the primers for next generation sequencing were added to both ends of sequence. Finally, DNA sequencing was completed as PE150 on a half lane of an Illumina Hiseq × Ten run.

Supernova assembler v2.1.1 (Zheng *et al.* 2016) (RRID:SCR_016756) was used to perform de novo genome assembly, initiated under the “supernova run” module after getting the raw genome sequence. The maximum reads, namely -maxreads, was set as “all.” The parameter of “-localcores” and “localmen” were set as 50 and 1024 GB based on K-MER analysis and previous experience to get a better result for Supernova run. Other parameters were set as default. Benchmarking Universal Single-copy Orthologs (BUSCO) v3.0.1 (Manni *et al.* 2021) (BUSCO, RRID:SCR_015008) and “vertebrate_odb10” data set from OrthoDB were used to assess the completeness of assembled genomes. In this process, 3354 essential genes of vertebrate were evaluated.

### Gene predication and functional annotation

We filtered several repetitive scaffolds caused by Supernova assembler with a “rm_dump_by_fa_seq.py” script. Both filtered genomes of *S. undulata* and *S. obscura* were treated with RepeatModeler v1.0.11 (Smit and Hubley 2008) using default parameters to find the repetitive sequences. The potential repeated protein-coding sequences are included in the database constructed by RepeatModeler. Therefore, the repeated sequences in database were aligned to perciform genes using blast. Subsequently, hits were removed from the repeat sequence database for further analyses. Based on the results of RepeatModeler, RepeatMasker v4.0.7 (Smit and Hubley 2013–2015) (Repeat Masker, RRID: SCR_012954) was used to mask the repetitive sequences in the genome.

All protein sequences of Percomorpha which are closely related to Sinipercidae were downloaded in “Taxonomy-Protein” option from NCBI for gene prediction. In addition, we accessed the full-length transcriptome data of *S. chuatsi* from NCBI (BioProject number: PRJNA552987) in order to conduct gene prediction. Blastn v2.7.1 (BLASTN, RRID: SCR_001598) was used to align the transcriptome to the genome (est2genome parameter in Maker). For more reliable alignments, blast hits were refined with Exonerate v2.2 (Slater and Birney 2005).

An iterative genome annotation was run for three times. Models used for gene prediction include de novo gene prediction, homology-based gene prediction and transcriptome-based prediction. In first run, Blastn v2.7.1 (BLASTN, RRID: SCR_001598) was used to map the transcriptome to the genome (est2genome parameter in Maker). For preliminary prediction, blast hits were refined with Exonerate v2.2 (Slater and Birney 2005). After alignment, MAKER v2.31.10 (Holt and Yandell 2011) (MAKER, RRID: SCR_005309) pipeline was used to merge the results for the first run of gene prediction. In the second run, the results of homology-based gene prediction and transcriptome-based prediction from the first run was used to train the gene prediction HMM mode of Augustus v3.3 (Stanke and Waack 2003) (Augustus, RRID: SCR_008417) base on *Danio rerio* (–species = zebrafish), and trained the SNAP (Korf 2004) HMM mode from scratch. The best gene model was chosen by MAKER subsequently. In third run, most parameters were set for the same as the second run, but “Re-annotation” option’s general feature format (GFF) file was used from the second run. To filter the predicted genes, we refined our data according to AED score (annotation edit distance). The gene with AED score <0.5 was retained. Well-annotated databases InterProScan5 (Jones *et al.* 2014) were applied to conduct the functional annotation. In this process, all the parameters were set as default and the mainstream databases including Pfam, GO in InterProscan5 were used for search. Finally, we uploaded the predicted genes to KAAS web server (https://www.genome.jp/kaas-bin/kaas_main) in bi-directional mode blast alignment for KEGG pathway annotation.

In addition, transposable sequences were annotated using LTR_harvest (Ellinghaus *et al.* 2008), LTR_Finder (Xu and Wang 2007), and LTR_retriever (Ou and Jiang 2018). The majority of RNA sequences were annotated using the cmscan program from infernal (Nawrocki and Eddy 2013). All annotated mRNAs were evaluated for noncoding RNA using ORF Length and GC content (LGC) (Wang *et al.* 2019).

### Gene family analysis

Protein data of 10 sibling species (*Collichthys lucidus, Nibea albiflora, Micropterus salmoide, Epinephelus lanceolatus, Etheostoma cragini, Sander lucioperca, Dissostichus mawsoni, Trematomus bernacchii, Cyclopterus lumpus, Anarrhichthys ocellatus*) were downloaded from ensembl (www.ensembl.org) to assess the annotated protein data of *S. undulata* and *S. obscura*. All 12 species were aligned with all-by-all blast. For blastp, “-seg” was set to filter the low complexity sequence. The sequences were clustered using Markov cluster algorithm (MCL) (Enright *et al.* 2002) with “-l 3” to determine the clustering granularity. The gene families that had a huge difference in copy number were trimmed.

OrthoFinder (Emms and Kelly 2019) was used to access the single-copy homologous genes of 12 species for a further phylogenetic relationship. Subsequently, we generated relevant species trees using iqtree (Chernomor *et al.* 2016) based on maximum likelihood method. The divergence time of *S. obscura* and *M. salmoide* from timetree (www.timetree.org) was used to deduce the ultrametric trees with r8s (Sanderson 2003). Finally, the change of gene families of the 12 species were inferred using computational analysis of gene family evolution (CAFE) (De Bie *et al.* 2006) with the clustering results of MCL and the ultrametric trees. Venn diagrams were printed using jvenn (Bardou *et al.* 2014) and Evolview (Subramanian *et al.* 2019) to demonstrate the rapid change of gene families for both *S. undulata* and *S. obscura*.

### Positive selection analysis

Phylogenetic analysis by maximum likelihood (PAML) was applied to test positive selection in genes of *S. undulata* and *S. obscura*. The longest isoform for all protein sequence and corresponding nucleotide sequence of both *S. undulata* and *S. obscura* from MAKER, and the 10 other species from Ensembl were obtained. Single-copy ortholgous genes were found using OrthoFinder. Mafft was applied to complete the alignment of the peptide orthogroups. All the coding sequences (CDS) were taken out and collected in a single file. The coding sequences were parsed to the peptide orthogroups by a custom script “get_same_name_seq_from_fasta.py” as the primary file for PAML. Subsequently, we removed the stop codons and end gaps in the CDS file. Seq ID in this file was also converted to respective species’ name in order to distinguish each other clearly. We reverse-translated peptide alignment into nucleotide sequence using trimAl. The end gaps within the nucleotide sequence were removed and the format of files was converted to phylip format for PAML analysis, with the phylogenetic tree generated above using single-copy homologous genes.

Detecting significance using chi2 Program included in PAML for selected orthogroups. Moreover, gene ontology (GO) and KEGG ID of positive selected gene were extracted. Based on selected genes, an enrichment analysis and a related graph were done with TBtools (Chen *et al.* 2020) on the background of all genes.

### Population history analysis

Pairwise sequentially Markovian coalescent (PSMC) analysis (Li and Durbin 2011) was carried out to get information about population history of both *S. undulata* and *S. obscura*. The 50× fastq data were mapped to the assembled genome of *S. undulata* and *S. obscura*, respectively, with the “BWA-MEM” algorithm from bwa v0.7.17 (Li 2013). SAMtools v0.1.19 (Li *et al.* 2009) was used to generate the diploid consensus with default settings, except for “-d 26 -D 160.” The default settings of PSMC were adopted except a generation time and a substitution rate of *Siniperca* were set as 2.5 years and $2.5\times 10^{-8}$ per site per year, respectively. The generation data was obtained from FishBase (Rainer *et al.* 2016). We chose the substitution rate from the study of Ray-finned fish (Takezaki 2018).

## Results and discussion

### Sequencing result, de novo genome assembly and evaluation

We obtained 586.99 million paired-end reads for *S. undulata* (88.1 Gbp data) and 625.09 million paired-end reads for *S. obscura* (93.8 Gbp data) after trimming low-quality reads. A 74.51 and 77.73% of nucleotides had a quality score greater than 30 in *S. undulata* and *S. obscura*, respectively, indicating reliable sequence data.

The average length of HMW DNA of *S. undulata* and *S. obscura* used for the construction of microfluidic partitioned DNA library were 43.28 and 34.43 kb, respectively. N50 read numbers of each barcode were 672 in *S. undulata* and 4020 in *S. obscura*. Average insertion sizes were 412 bp in *S. undulata* and 397 bp in *S. obscura*. Average distance between SNPs were 1050 bp in *S. undulata* and 406 bp in *S. obscura*.

The de novo genome assembly of *S. undulata* and *S. obscura* were processed with 88.1 G and 93.8 G of sequencing data, respectively. The final genome size of *S. undulata* and *S. obscura* were 744 and 733 Mb. From the genome assembly, we gained a total of 13,658 scaffolds in *S. undulata* and 16,412 in *S. obscura*. For *S. undulata*, 216 scaffolds were larger than 1 Mb, accounting for 58.17% of the whole genome. For *S. obscura*, 188 scaffolds were larger than 1 Mb, accounting for 75.61% of the whole genome. The contig N50 of the genome of *S. undulata* and *S. obscura* were 45.6 and 36.56 kb, respectively; the scaffold N50 were 1.34 and 2.75 Mb, respectively. The longest scaffolds of *S. undulata* and *S. obscura* were 8.37 and 11.44 Mb. G and C deoxyribonucleic acid content of the genomes were 40.36 and 40.34% (Table 1).

**Table 1.** Statistics of the genome assemblies of *Siniperca undulata* and *S. obscura*.

Metric	*S. obscura*	*S. undulata*
Assembly size (Mb)	733	744
Scaffolds count	16,412	13,458
Scaffolds count (>=1 Mb)	188	216
>= 1 Mb scaffolds ratio (%)	75.61	58.71
Max length scaffold (Mb)	11.44	8.37
Scaffold N50 (Mb)	2.75	1.34
Contig N50 (kb)	36.56	45.6
GC (%)	40.34	40.36
BUSCO (%)	93.1	93.7

BUSCO v3.0.1 (BUSCO, RRID:SCR_015008) were processed for genome integrity assessment, using 3353 genes of vertebrates. For *S. undulata*, 3141 genes (93.7%) were completely matched in the assembled genome, containing 3119 (93.0%) single-copy genes and 22 (0.7%) multicopy genes. In addition, 140 (4.2%) genes were fragmented and 73 (2.1%) were not found. For *S. obscura*, 3121 genes (93.1%) were completely matched in the assembled genome, containing 3092 (92.2%) single-copy genes and 29 (0.9%) multicopy genes. One hundred and fifty (4.5%) genes were fragmented and 83 (2.4%) were not found. The final results indicate that most BUSCO genes were fully assembled in the genome of both *S. undulata* and *S. obscura*. This result showed our genomes contiguity is low, but gene completeness is good. This quality meets the needs of our analysis.

### Gene prediction and functional annotation

There were 24,358 genes predicted for *S. undulata*, and 24,360 genes predicted for *S. obscura* (Supplementary Material). The existing databases, including KEGG and InterProScan5, were used to evaluate the predicted coding genes, indicating that 95.8 and 93.6% of the genes had at least one hit in *S. undulata* and *S. obscura*, respectively. The results of blast on several databases are shown in Table 2.

**Table 2.** Annotation using InterProscan.

Metric	*S. obscura*	*S. undulata*
Gene number	24,360	24,358
Annotated gene number	22,818	23,344
Annotation ratio	93.6%	95.8%
GO	16,373/67.2%	16,562/67.9%
SuperFamily	16,158/66.3%	16,281/66.8%
Pfam	19,692/80.8%	19,544/80.2%
ProSiteProfiles	12,085/49.6%	12,008/49.2%
SMART	11,005/45.1%	10,934/44.8%
Gene3D	17,322/71.1%	17,431/71.5%
PANTHER	21,409/87.8%	21,743/89.2%
Coils	5,317/21.8%	5,525/22.6%
PRINTS	5,686/23.3%	5,408/22.2%
CDD	8,791/36%	8,621/35.5%
ProSitePatterns	6,203/25.4%	5,943/24.3%
PIRSF	1,313/5.38%	1,105/4.53%

We found the *S. obscura* genome contains a total of 26.5% repeat sequences, of which classified DNA transposons account for 8.23% of the genome, while the *S. undulata* genome contains a total of 27.41% repeat sequences, of which classified DNA transposons account for 8.70% of the genome. All repeat database in Supplementary Material. In addition, data such as tRNA and ncRNA obtained using Infernal and LGC also available in Supplementary Material.

### Gene family analysis

**Fig. 2.**
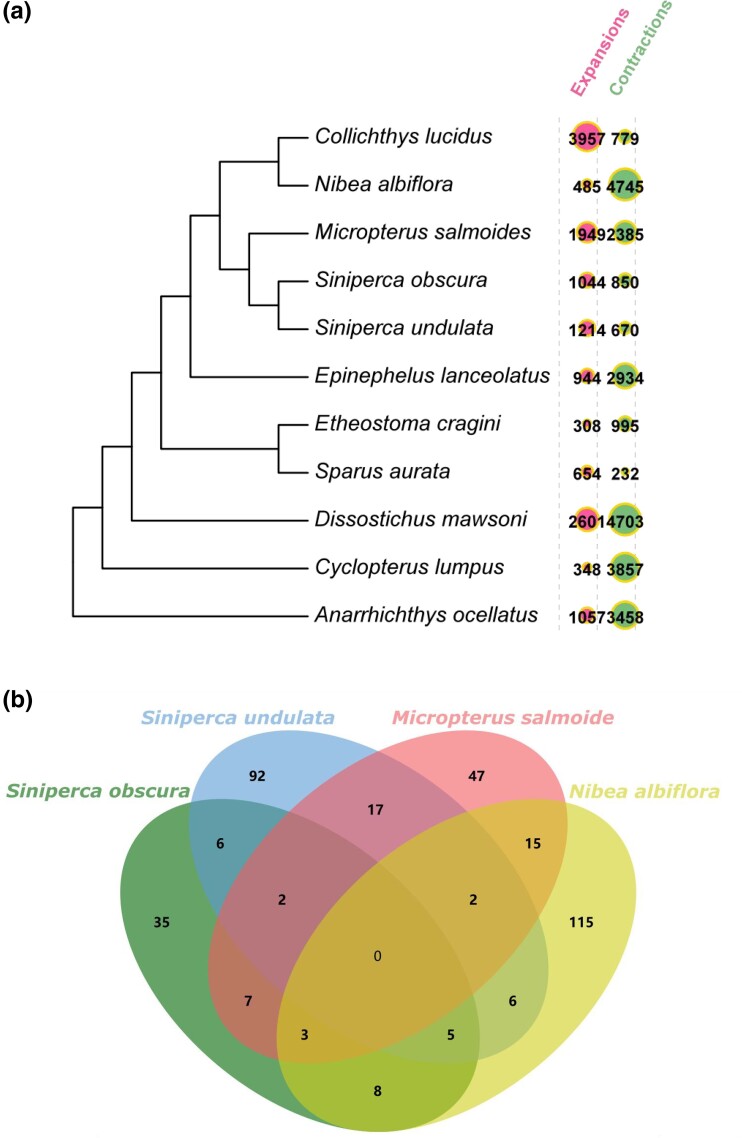
a) Tree for all species with labeling of expansion, contraction, rapidly evolve gene families. b) A Venn diagram of rapidly evolving gene families for *Siniperca undulata*, *S. obscura*, *Micropterus salmoide*, and *Nibea albiflora*.

The results of gene family analysis in *S. undulata* and *S. obscura* are shown in Fig. 2. For *S. undulata*, there were 88 rapidly evolving gene families, 1616 expanding gene families, and 1392 contracting gene families. For *S. obscura* were 59, 1200, and 1423, respectively. More gene families contracted and less gene families expanded in *S. obscura* compared with *S. undulata*. *Siniperca undulata* has a wider geographic distribution than *S. obcura*. *Siniperca undulata* has adapted to a variety of environments, indicating that they may face stronger selective pressure than *S. obscura* did, which led to more expansion of gene families in *S. undulata*. And *S. obscura* have more gene family contractions, may suffer more purifying selections. In addition, the Venn diagrams have no overlap between any rapidly expanding or contracting gene families in *S. undulata* and *S. obscura* (Fig. 2).

Search genes function for rapidly gene families result, we found that gene families related to metabolism, growth rate, motility, and immunity evolving rapidly in *S. undulata* and *S. obscura*. Among them, metabolism-related gene families expanded in *S. obscura* and contracted in *S. undulata*. Growth rate-related gene families expanded in *S. undulata* and contracted in *S. obscura*. Motility-related gene families expanded in *S. undulata* and contracted in *S. obscura*. For immunit, some expanded in *S. obscura* and contracted in *S. undulata*, and others expanded in *S. undulata* and contracted in *S. obscura*.

### Positive selection analysis

Search genes function for most significant PAML analysis result showed that genes related to growth, athletic ability and immunity were positively selected in both *S. undulata* and *S. obscura*. In *S. undulata*, positive selected genes were associated with growth and locomotion. This result was cooperated by the gene family analysis. In addition, some positive selection genes in *S. undulata* are associated with cancer formation.

**Fig. 3.**
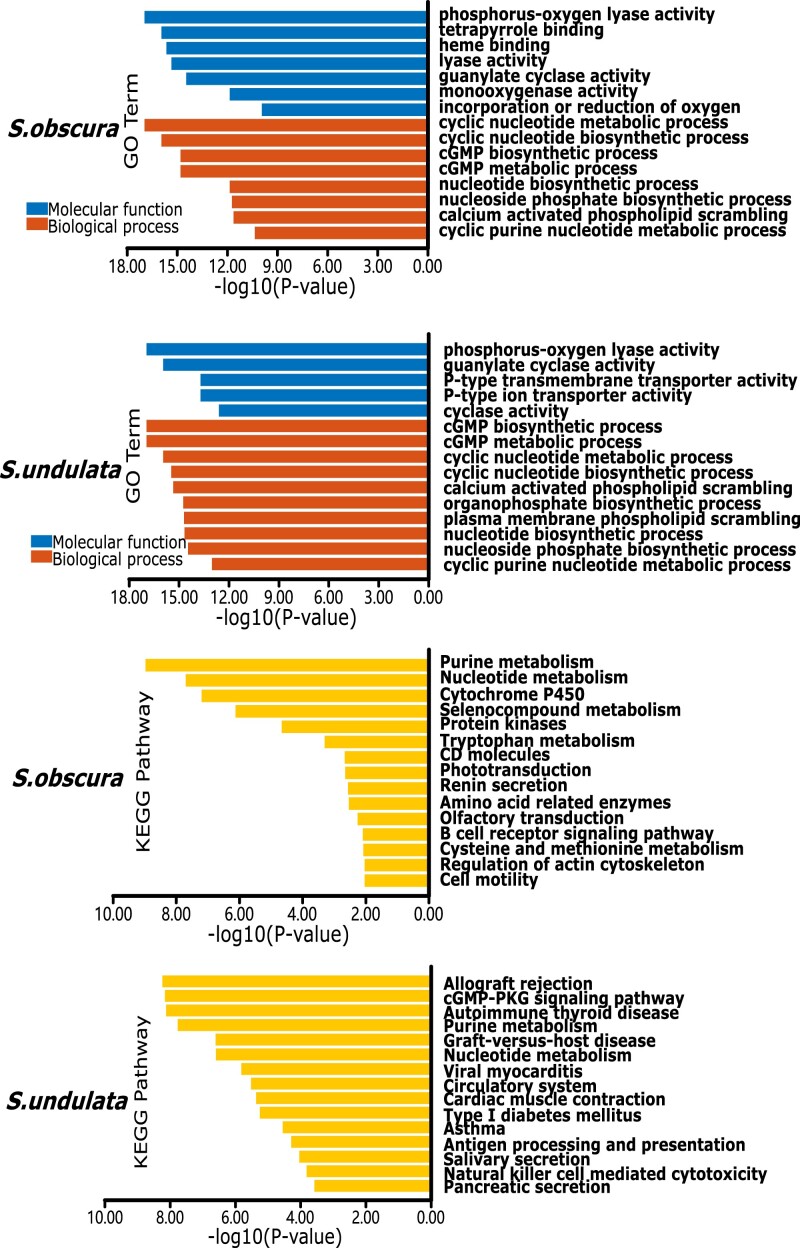
Enrichment scores of GO terms and KEGG pathway in *Siniperca undulata* and *S. obscura*. The above two figures show the results of GO enrichment analysis for two species, and the following two figures show the results of KEGG enrichment analysis. The results show significant enrichment in phosphorus–oxygen lyase activity and purine metabolism for both species.

We performed GO enrichment analysis and KEGG enrichment analysis using all positive selection genes. In GO term, phosphorus–oxygen lyase activity was enriched in both *S. obscura* and *S. undulata*. Phosphorus–oxygen lyase enzymes catalyze the cleavage of a phosphorus–oxygen bond, which can release large quantities energy in organisms. cGMP biosynthetic process was enriched in *S. obscura* and *S. undulata* as biological process, which is involved in the formation of cyclic GMP. Other enriched molecular functions and biological processes of *S. obscura* and *S. undulata* are shown in Fig. 3. In KEGG pathway, purine metabolism was enriched in *S. obscura* and *S. undulata*, which maintains cellular pools of adenylate and guanylate via synthesis and degradation of purine nucleotides (Yin *et al.* 2018). Autoimmune thyroid disease was enriched in *S. undulata*, which is a pathway related to an autoimmune disorder affecting the thyroid gland. Cytochrome P450 was enriched in *S. obscura*, which is important for xenobiotic metabolism and steroid transformation (Munro *et al.* 2018). All results are included in the Supplementary Material.

### Population history

**Fig. 4.**
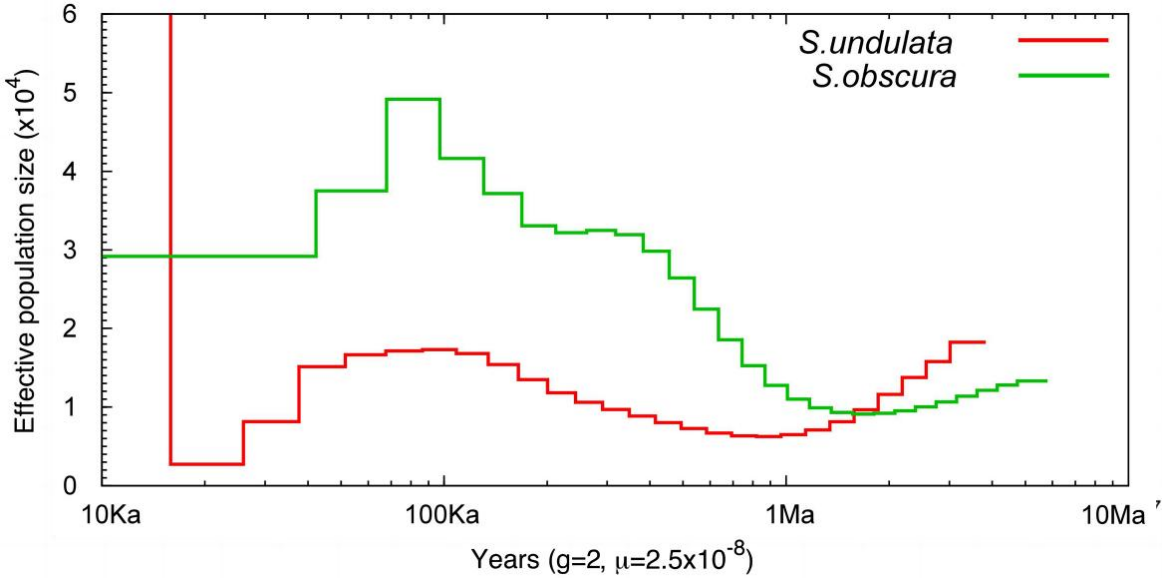
PSMC analysis for *S. undulata* and *S. obscura* (10 Ma–10 Ka). This graph shows the population history of two species during the period from 10 Ma to 10 Ka. The *x*-axis is a logarithmic scale with markings from left to right corresponding to four historical time points: 10 Ka, 100 Ka, 1 Ma, and 10 Ma. The *y*-axis represents effective population size.

The fluctuation of effective population size of *S. undulata* and *S. obscura* is shown in Fig. 4. From 4 to 2 Ma, the population size of both *S. undulata* and *S. obscura* was declining; among which the downward trend of *S. undulata* was more significant. This phenomenon may be attributed to the Quaternary glaciation among which the global temperature dropped rapidly and the world entered an ice age, leading to the decline of the two species. The population size of *S. obscura* started rising from 2 Ma, probably adapted to environmental changes rapidly due to their smaller body size. Compared with *S. obscura*, *S. undulata* is larger indicating a higher energy demands.

Between 90 and 70 Ka, the population sizes of both *S. undulata* and *S. obscura* showed a rising trend, which may be caused by the extension of the last interglacial period (Cui *et al.* 2011), the warmest period since 1.5 Ma. After this phase, a descent phase began from 70 to 20 Ka, which is in accordance with the Tali glacial period in eastern China (57–16 Ka) (Wan *et al.* 2011). Due to the lack of specific mutation rate data for this species, our estimates may contain errors. However, the PSMC algorithm mentioned in the article is relatively accurate for the time period mentioned, previous PSMC analysis results for *S. knerii* (Lu and Zhao 2020a) with the geological history of China. In addition, the comparative analysis of the population history of these two species under the same parameters should be relatively reliable.

### Similarities and differences of selected genes

We found that several gene families related to growth expanded in *S. undulata* and contracted in *S. obscura*. In these gene families, we identified that the dner, CDH10, and nrxn2b genes were associated with growth. The dner gene mainly regulates the expression of epidermal growth factor (Wouters *et al.* 2005). The CDH10 gene is important for proliferation, differentiation, and migration (Alattia *et al.* 1999). The nrxn2b gene is essential for a series of biological processes in cell growth (Sasaki *et al.* 2004). Furthermore, many expanding gene families in *S. undulata* include Tenm1 and Vwdel genes. The Tenm1 gene can promote the generation of synapses among neural cells and connect neurons (Li *et al.* 2018), while the Vwdel gene regulates proliferation and differentiation of enamel epithelial cells in dental tissue (Iwata *et al.* 2022), and its expression is associated with the regeneration of lambs and fins (Leigh *et al.* 2020). Additionally, the rnf183 gene, which takes part in the regulation of the cell cycle and the promotion of terminal differentiation in the cell cycle (Lu *et al.* 2020b), and the ltn1 gene, which plays an important role in the growth of the embryo (Yang *et al.* 2021), both positively selected in *S. undulata*. The positive selection genes in *S. undulata* corroborates our findings about the growth-related differences in the results of gene family analysis.

Several gene families related to athletics and metabolism show expansion in *S. obscura* but contraction in *S. undulata*. The gbf1 gene-related gene family, which expands in *S. obscura*, regulates the expression of ADP-ribosylation factor (ARF) (Abergel *et al.* 1998). ARF is involved in material transportation and signal transduction (Muthamilarasan *et al.* 2016), leading to frequent material exchange and synthesis, which may indicate a faster metabolism rate in individuals. In addition, the exonuclease 3–5′ domain containing 2 (exd2) gene participates in DNA double-strand break repair and functions as a ribonuclease for the regulation of mitochondrial translation (Park *et al.* 2019) The diaphanous-related formin 1 (diaph1) and diaphanous-related formin 3 (diaph3) genes are both linked to athletic ability, and they show expansion in *S. undulata* but contraction in *S. obscura*. The main function of DIAPH1 is to promote the combination of guanosine triphosphatase and actin, stabilize microtubules, and promote cell migration (Zaoui *et al.* 2010). DIAPH1 also induces the collection of actin to generate new filaments, facilitating muscle formation. Similar to diaph1, the diaph3 gene can promote the reconstruction of the cytoskeleton and the formation of stress fibers (Tanaka 2000).

Different immune genes under selection in *S. obscura* and *S. undulata*. The C1gtnf gene, which is associated with immunity, expand in *S. obscura* and contract in *S. undulata*, which is involved in autoimmunity, cancer, and other pathological diseases (Kishore and Reid 2000). The C1gtnf gene also involves a tumor necrosis factor-like domain, whose main function is to inhibit the growth of tumor cells and strengthen the ability of anti-infection and individual immunity (Sessler *et al.* 2013). Sema3fb and H2-L genes are also related to immunity, expand in *S. undulata* and contract in *S. obscura*. The Sema3fb gene mainly regulates a series of immune globulin that have diverse functions, including antigen binding, enhancing immunity, and resisting infection (Schroeder 2010). The H2-L gene regulates major histocompatibility complex class I chain-related molecules (MIC) in organisms. MIC mainly expresses in gastric epithelial cells, endothelial cells, and fibroblasts, activating the natural killer cell receptor (NKG2D), which takes part in the recognition of virus-infected cells and killing tumor cells (Collins 2004). Additionally, Sox5 and Sox8 genes take part in the immune response, positively selected in *S. undulata*. The expression of Sox5 may be highly related to the development of tumors, the size of focal infection, and lymphatic metastasis (Hock *et al.* 2007). Sox8 gene belongs to the SOX gene family, which takes part in the formation of tumors and cancer as a transcription factor (Castillo and Sanchez-Cespedes 2012). The Btbd6b gene positively selected in *S. undulata* and not in *S. obscura*. This gene has a close relationship with Cul3. As a tumor suppressor gene, Cul3 encodes a protein named Cullin-3, which plays an important role in fighting against cancer (Canning *et al.* 2013).

### Adaptive evolution hypothesis for *S. undulata* and *S. obscura*

By comparing the differences in selected genes between *S. undulata* and *S. obscura*, we found that differences in growth, metabolism, vitality, and immunity reflect the differences in adaptive evolution between two species. Based on these results, we propose the following hypotheses.

Firstly, in terms of growth, development, and movement ability, in *S. undulata*, the expansion of gene families related to the dner and Vwdel genes may be associated with the golden wavy pattern on the skin of *S. undulata*. Gene families related to CDH10, nrxn2b, Tenm1, diaph1, and diaph3 are related to neural development, muscle development, and cell proliferation and differentiation, and these gene families have expansion in *S. undulata*, which may led to increase body size and activity ability.

Smaller species have a larger surface area, which leads to a faster exchange of material and energy with the surrounding environment. In *S. obscura*, the expansion of gene families related to the Gbf1 and exd2 genes has been observed, which are related to substance transport, signal transduction, and DNA repair. Combined with the small body size of *S. obscura*, this may be the result of its adaptation to a faster metabolic rate.

In terms of immunity, we found that the C1gtnf gene family had expansion in *S. obscura*. This gene has the main function of inhibiting tumor cell growth and enhancing immunity. However, in *S. undulata*, gene families related to Sema3fb and H2-L have expansion, while the Sox5, Sox8, and Btbd6b genes have positive selection in *S. undulata*. These genes are not only related to antigen binding, resistance to infection, inhibition of cancer, and activation of natural killer cells but also involved in the formation of tumors. Based on these results, we speculate that there may be more carcinogenic and pathogenic factors in the habitat of *S. undulata*, leading to more relevant genes being under selection in this species.

## Supplementary Material

jkad101_Supplementary_Data

## Data Availability

The raw data are deposited in NCBI with SRA accessions numbers: SRR22544051-SRR22544058 for *S. undulata* and SRR22560318-SRR22560324 for *S. obscura*. The BioSample is available with accession number SAMN31858682 for *S. undulata* and SAMN31942353 for *S. obscura* at NCBI. Two assembled genomes are available at NCBI BioProject, PRJNA904940 for *S. undulata* and PRJNA906929 for *S. obscura*, and genomes also available in figshare http://doi.org/10.6084/m9.figshare.21732578. The *S. chuatsi* full-length transcriptome data were downloaded from NCBI and the BioProject number is PRJNA552987. Supplementary Material and script, annotation data available at figshare: http://doi.org/10.6084/m9.figshare.21709745 (for gene annotation), http://doi.org/10.6084/m9.figshare.21709703 (for script), https://doi.org/10.6084/m9.figshare.22494340(for Repeat, LTR and RNA annotation) and https://doi.org/10.6084/m9.figshare.21731762 (for GO & KEGG enrichment analysis result). Supplemental material.
